# Endogenous ω-3 Fatty Acid Production by *fat-1* Transgene and Topically Applied Docosahexaenoic Acid Protect against UVB-induced Mouse Skin Carcinogenesis

**DOI:** 10.1038/s41598-017-11443-2

**Published:** 2017-09-14

**Authors:** Hye-Won Yum, Jin Park, Hyun-Jung Park, Jun Wan Shin, Yong-Yeon Cho, Su-Jung Kim, Jing X. Kang, Young-Joon Surh

**Affiliations:** 1 Tumor Microenvironment Global Core Research Center, College of Pharmacy, Seoul National University, Seoul, 08826 South Korea; 2 Research Institute of Pharmaceutical Sciences, College of Pharmacy, Seoul National University, Seoul, 08826 South Korea; 3 Department of Molecular Medicine and Biopharmaceutical Sciences, Graduate School of Convergence Sciences and Technology, Seoul National University, Seoul, 08826 South Korea; 4 Cancer Research Institute, Seoul National University, Seoul, 03080 South Korea; 5 Integrated Research Institute of Pharmaceutical Sciences, College of Pharmacy, The Catholic University of Korea, Bucheon, Gyeonggi-do 420-743 South Korea; 6 Laboratory for Lipid Medicine and Technology, Massachusetts General Hospital, Harvard Medical School, Boston, MA 02129 USA

## Abstract

The present study was intended to explore the effects of endogenously produced ω-3 polyunsaturated fatty acids (PUFAs) on ultraviolet B (UVB)-induced skin inflammation and photocarcinogenesis using hairless *fat-1* transgenic mice harboring ω-3 desaturase gene capable of converting ω-6 to ω-3 PUFAs. Upon exposure to UVB irradiation, *fat-1* transgenic mice exhibited a significantly reduced epidermal hyperplasia, oxidative skin damage, and photocarcinogenesis as compared to wild type mice. The transcription factor, Nrf2 is a master regulator of anti-inflammatory and antioxidant gene expression. While the protein expression of Nrf2 was markedly enhanced, the level of its mRNA transcript was barely changed in the *fat-1* transgenic mouse skin. Topical application of docosahexaenoic acid (DHA), a representative ω-3 PUFA, in wild type hairless mice induced expression of the Nrf2 target protein, heme oxygenase-1 in the skin and protected against UVB-induced oxidative stress, inflammation and papillomagenesis. Furthermore, transient overexpression of *fat-1* gene in mouse epidermal JB6 cells resulted in the enhanced accumulation of Nrf2 protein. Likewise, DHA treated to JB6 cells inhibited Nrf2 ubiquitination and stabilized it. Taken together, our results indicate that functional *fat-1* and topically applied DHA potentiate cellular defense against UVB-induced skin inflammation and photocarcinogenesis through elevated activation of Nrf2 and upregulation of cytoprotective gene expression.

## Introduction

Skin is repeatedly exposed to various external stressors, such as solar radiation and microbes^1,2^. In response to changing external and internal environment, the skin produces cytokines, neurotransmitters, neuroendocrine hormones and their corresponding receptors^3–5^. These capabilities of skin allow it to preserve the homeostasis which is critical for organism fitness and survival.

Exposure of skin to excessive ultraviolet B (UVB) causes the production of reactive oxygen species (ROS), which results in massive infiltration of inflammatory cells, especially, neutrophils at the site of tissue injury^6^. ROS play important roles in the modulation of intracellular signal transduction involved in each stage of photocarcinogenesis, i.e., initiation, promotion, and progression^7^. One of the key enzymes mediating inflammatory signal transduction is cyclooxygenase-2 (COX-2) that catalyzes the rate-limiting step in the biosynthesis of prostaglandins. Nuclear factor kappa B (NF-κB) and signal transducer and activator of transcription 3 (STAT3) are two representative redox-sensitive transcription factors responsible for regulating COX-2 expression^8–14^. A battery of antioxidant enzymes play a central role in counteracting excessive ROS accumulation, thereby maintaining the cellular redox balance. Examples are heme oxygenase-1 (HO-1) and NAD(P)H:quinone oxidoreductase 1 (NQO1). The proximal promoter regions of genes encoding HO-1 and NQO1 harbor a consensus sequence known as antioxidant response element (ARE) which is a preferred binding site of nuclear factor-erythroid related factor-2 (Nrf2)^15^.

Long chain ω-3 polyunsaturated fatty acids (ω-3 PUFAs) abundant in fish oil have been reported to retain marked anti-inflammatory and anti-oxidative properties, which contribute to their chemopreventive potential^16,17^. In contrast, ω-6 PUFAs have pro-inflammatory activities. The ω-6/ω-3 PUFA ratio has been considered essential for determining the risk for some human malignancies^18–21^. Kang *et al*. developed genetically engineered mice of C57BL6 background carrying a *fat-1* gene which encodes an ω-3 fatty acid desaturase derived from *Caenorhabditis elegans* ^22,23^. These transgenic mice are capable of converting ω-3 PUFAs from ω-6 PUFAs, thereby maintaining a significantly elevated ω-3/ω-6 PUFA ratio in their tissues and organs.

In the present study, hairless *fat-1* transgenic mice were generated by cross-breeding of male *fat-1* ^+/−^ mice with female SKH-1 hairless mice. By utilizing these hairless *fat-1* transgenic mice maintained on ω-6 PUFA containing diet, we investigated the effect of endogenously formed ω-3 PUFAs on the development of UVB-induced tumors as well as oxidative stress and inflammation in the skin.

## Results

### Generation of hairless *fat-1* mice and their skin PUFA profile

**Figure 1:**
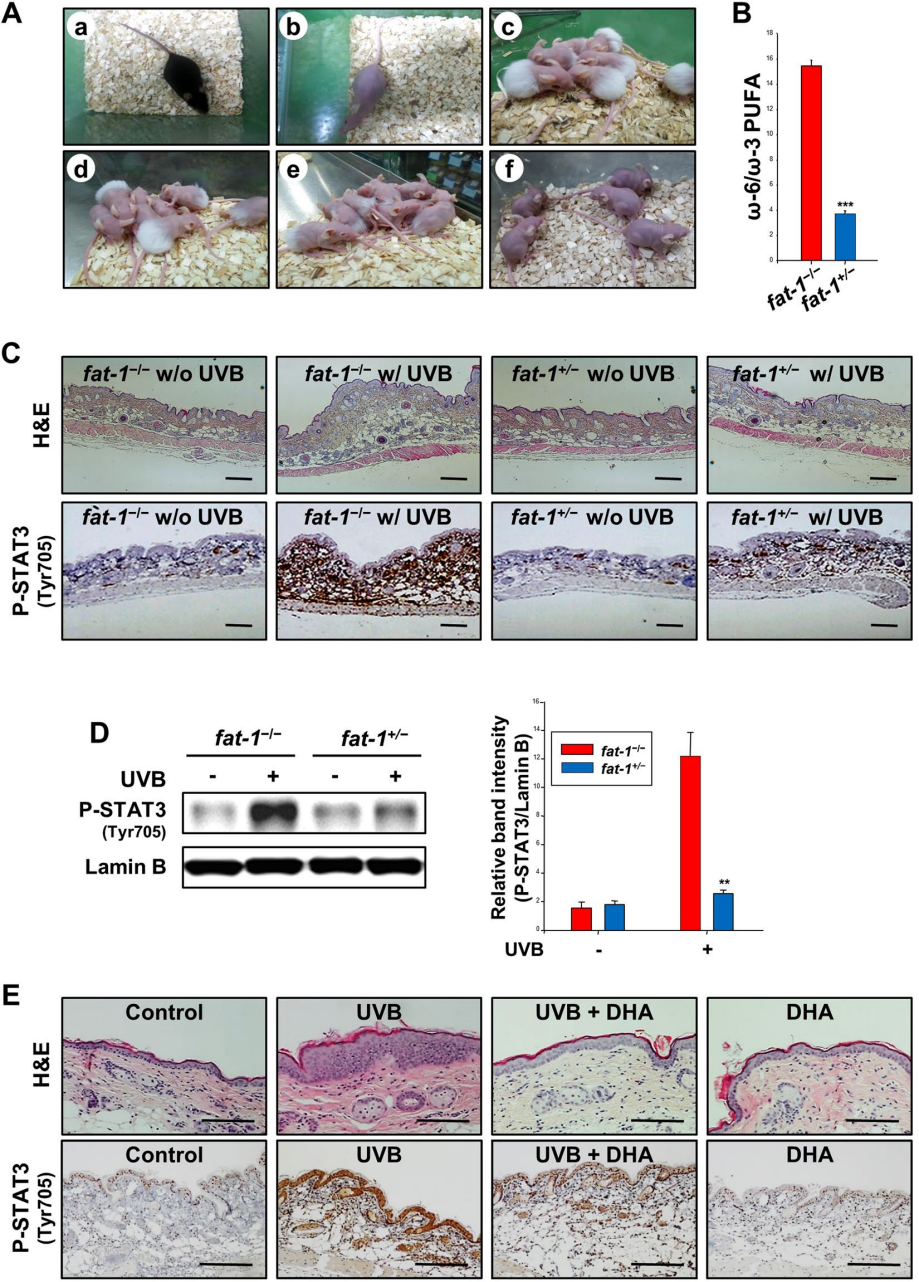
UVB-induced inflammation was ameliorated in *fat-1* transgenic and DHA-treated mouse skin. (**A**) *fat-1* ^+/−^ haired mice (a) were crossed with SKH-1 hairless mice (b) to generate *fat-1* ^−/−^ and *fat-1* ^+/−^ progenies. Hairless *fat-1* ^−/−^ and *fat-1* ^+/−^ mice during (c–e) and after (f) first hair cycle. After an initial hair cycle, the skin became eventually hairless, suitable for subsequent studies undertaken. (**B**) Both hairless *fat-1* ^−/−^ and *fat-1* ^+/−^ littermates born to the same mother were maintained on a diet (10% safflower oil) high in ω-6 and scarce in ω-3 PUFAs. After the dietary regimen, the PUFA profiles of mouse skin were analyzed by gas chromatography. Significantly elevated levels of ω-3 vs. ω-6 fatty acids were observed in skin tissues of hairless *fat-1* ^+/−^ mice as compared to those of hairless micelacking the *fat-1* gene. Data are expressed as means ± SE. ***p < 0.001 versus WT mice. Newly developed female hairless *fat-1* as well as WT mice (*n* = 5 per each treatment group) were irradiated on their backs with UVB light (180 mJ/cm^2^) for 2 h. (**C**) Skin thickness in hairless *fat-1* transgenic and WT mice was visualized by H&E staining at 2 h following UVB exposure. Formalin-fixed and paraffin-embedded tissues from UVB-irradiated mice were also immunostained for phospho-STAT3 (Tyr^705^), and counterstained with hematoxylin. Magnifications, ×100. Scale bar is 100 µm. (**D**) Nuclear extracts from different groups were subjected to SDS-PAGE and immunoblotted to detect phosphorylated forms of STAT3 (Tyr^705^). **p < 0.01 versus UVB-irradiated WT mice. (**E**) Dorsal skin of female HR-1 hairless mice (*n* = 5 per treatment group) were treated topically with DHA (10 µmol) 30 min before UVB (180 mJ/cm^2^) irradiation. Mice were sacrificed after 2 h of UVB irradiation. Irradiated skin tissue sections were stained with H&E to examine inflammatory changes resulting in increased skin thickness. Control animals were treated with acetone alone and left unirradiated. The sections of skin tissues were also subjected to immunohistochemical analysis of phosphorylated of STAT3 at Tyr^705^. Magnifications, ×200. Scale bar is 100 µm.

To avoid the inconvenience of frequent hair removal in determining whether an increased ω-3 PUFA tissue accumulation in *fat-1* mice is protective against skin carcinogenesis induced by repeated UVB irradiation, we generated hairless *fat-1* mice by crossing *fat-1* ^+/−^ haired and SKH-1 hairless mice (Fig. 1A). Thus, the *fat-1* transgenic mice of the C57BL6 strain were subsequently backcrossed five times to SKH-1 hairless mice. The *fat-1* ^+/−^ animals were segregated for the hairless phenotype. The resulting mice had the predominant genetic background of Skh:hr-1 hairless (>96.875%). All the experiments were performed by comparing mice of the same genetic background with or without carrying the *fat-1* transgene. The hairless mutation showed normal development of the first hair coat in the first hair cycle. Starting at the 2 week of age, they lost their hair coat rapidly^24^. At weaning, they were completely hairless. Both hairless *fat-1* ^−/−^ and *fat-1* ^+/−^ littermates born to the same mother were maintained on an identical diet containing ω-6 PUFAs, but deficient in ω-3 ones. Analysis of the total lipids extracted from skin tissues of hairless *fat-1* ^+/−^ mice showed a PUFA profile distinct from that of hairless *fat-1* ^−/−^ (Fig. 1B and Table 1). There were significantly lower levels of ω-6 PUFAs and much higher proportions of ω-3 PUFAs in the skin of hairless *fat-1* ^+/−^ mice compared to those *fat-1* ^−/−^ mouse skin. The ratios of the ω-6 (18:2n-6, 18:3n-6, 20:3n-6 and 20:4n-6) to the ω-3 PUFAs (18:3n-3, 20:3n-3, 20:5n-3 and 22:6n-3) in skin tissues of hairless *fat-1* ^−/−^ and *fat-1* ^+/−^ mice were 15.48:1 and 3.77:1, respectively. This indicates that the transgene is functionally active and transmittable.

**Table 1:** PUFA composition in the skin of hairless *fat-1* transgenic and WT mice.

		WT	*fat-1* ^+/−^
Linoleic acid	C18:2	31.81 ± 0.62	27.06 ± 0.86^a^
γ-Linolenic acid	C18:3	0.46 ± 0.06	0.22 ± 0.01^a^
Dihomo-γ-linolenic acid	C20:3	1.03 ± 0.04	0.54 ± 0.06^b^
Arachidonic acid	C20:4	0.37 ± 0.02	0.04 ± 0.01^c^
ω-6 PUFAs (%)		33.66 ± 0.60	27.86 ± 0.89^b^
α-Linolenic acid	C18:3	0.90 ± 0.04	2.46 ± 0.13^c^
Eicosatrienoic acid	C20:3	0.56 ± 0.06	1.03 ± 0.01^b^
Eicosapentaenoic acid	C20:5	0.35 ± 0.04	1.29 ± 0.02^c^
Docosahexaenoic acid	C22:6	0.36 ± 0.01	2.63 ± 0.11^c^
ω-3 PUFAs (%)		2.18 ± 0.03	7.41 ± 0.24^c^
ω-6/ω-3 PUFAs		15.48 ± 0.48	3.77 ± 0.22^c^
ω-3/ω-6 PUFAs		0.06 ± 0.004	0.27 ± 0.02^c^

The PUFA composition in the skin of hairless *fat-1* and WT mice was measured as described in Methods. Data are expressed as means ± SE. (*n* = 3); ^a^ *p* < 0.05, ^b^ *p* < 0.01 and ^c^ *p* < 0.001 versus hairless WT mice.

### UVB-induced acute skin inflammation was ameliorated in *fat-1* transgenic and DHA-treated mice

The thickness of the non-keratinized epidermis was decreased in the skin of short-term UVB-irradiated hairless *fat-1* mice compared with wild type (WT) hairless littermates (Fig. 1C, *upper*). Persistently activated STAT3 plays an important role in photocarcinogenesis through upregulation of genes involved in tumor-associated inflammation, anti-apoptosis and proliferation^25^. One of the essential events in activation of STAT3 signaling is phosphorylation at its tyrosine 705 residue (Tyr^705^). As shown in Fig. 1C (*lower*), WT hairless mice irradiated with UVB exhibited markedly increased expression of P-STAT3 (Tyr^705^) which appeared as brown color staining. A significantly reduced proportion of phosphorylated STAT3-positive cells was observed in *fat-1* transgenic mouse skin irradiated with UVB for 2 h. *fat-1* mouse skin also showed markedly decreased nuclear translocation of P-STAT3 after UVB irradiation compared with WT mouse skin (Fig. 1D). Topical application of DHA also ameliorated UVB-induced epidermal hyperplasia (Fig. 1E, *upper*) and attenuated UVB-induced expression of P-STAT3 (Tyr^705^) in WT hairless mice (Fig. 1E, *lower*).

### UVB-induced skin tumor development was suppressed in hairless *fat-1* mice compared with that in WT mice

**Figure 2:**
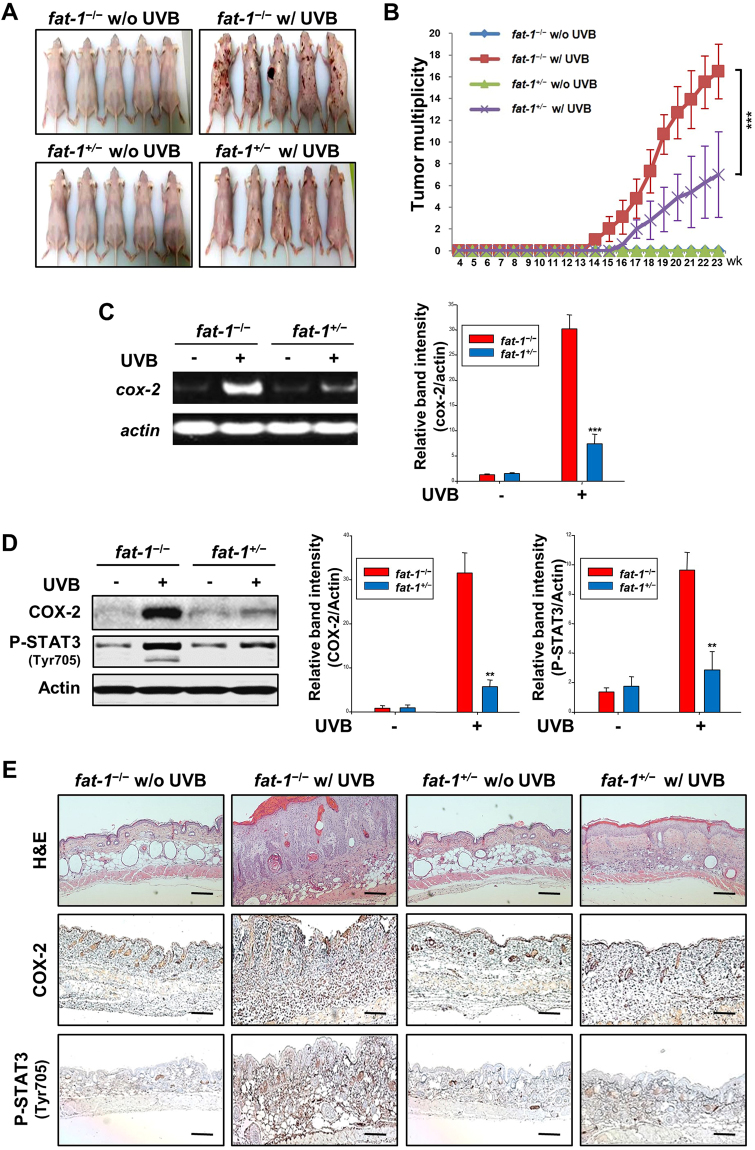
UVB-induced skin tumor development was suppressed in hairless *fat-1* mice compared with that in WT mice. The female hairless *fat-1* or WT mice (*n* = 10 per group) were irradiated with UVB (180 mJ/cm^2^) three times a week until termination of the experiment. (**A**) The photographic images represent papillomas formed at the 23^rd^ week of UVB treatment. (**B**) Starting four week following UVB treatment, tumors of at least 1 mm diameter were counted every week till the 23^rd^ weeks. The results were expressed as the average number of papillomas per mouse. Results are expressed as means ± SD. ***p < 0.001 versus the UVB-irradiated WT mice. (**C**) Total RNA was isolated from skin tissues, and mRNA expression of *cox-2* was examined by RT-PCR analysis. (**D**) Whole surrounding tissue extracts (30 μg protein) were analyzed for the expression of COX-2 and P-STAT3 (Tyr^705^) by immunoblotting. Data are expressed as means ± SE. **p < 0.01 and ***p < 0.001 versus UVB-irradiated WT mice. (**E**) H&E staining of papillomas showed hypertrophic squamous epithelium forming papillary fronds. The sections of skin tissues were also subjected to immunohistochemical analysis of COX-2 and P-STAT3 (Tyr^705^). Positive COX-2 and P-STAT3 (Tyr^705^) staining yielded a brown-colored product. Magnifications, ×100. Scale bar is 100 µm.

After repeated UVB irradiation thrice a week for 23 weeks, representative photographs of mice from different groups were taken. Figure 2A shows that the tumor burden in the hairless *fat-1* mice was much lower than that in the hairless WT mice. The tumor multiplicity between the two experimental groups was significantly different (Fig. 2B). In the hairless *fat-1* mice, the average number of tumors per mouse was 7 compared with 16.5 in the WT littermates.

### UVB-induced expression of COX-2 and phosphorylation of STAT3 were diminished in the papillomas of hairless *fat-1* mice compared with those in the WT mice

Inappropriate upregulation of COX-2 induces malignant changes in epidermal keratinocytes^26^ and prolongs the survival of malignant or transformed cells with metastatic potential^27^. STAT3 is a well known transcription factor involved in the regulation of COX-2 expression^13^. A significant decrease in expression of COX-2 at both transcriptional (Fig. 2C) and translational (Fig. 2D) levels was noted in the papillomas of the hairless *fat-1* mice. Concomitant with downregulation of COX-2, the level of P-STAT3 (Tyr^705^) was also markedly decreased in *fat-1* mouse skin irradiated with UVB (Fig. 2D). The above findings were also verified by hematoxylin and eosin (H&E) staining and immunohistochemical analysis. The thickening of epidermal layer and inflammatory cell infiltration in WT mouse skin caused by long-term UVB exposure were reduced in *fat-1* skin (Fig. 2E, *upper*). Likewise, expression of COX-2 (Fig. 2E, *middle*) and P-STAT3 (Fig. 2E, *lower*) in the skin of *fat-1* mice was substantially abrogated.

### DHA inhibited UVB-induced papillomagenesis in hairless mouse skin

**Figure 3:**
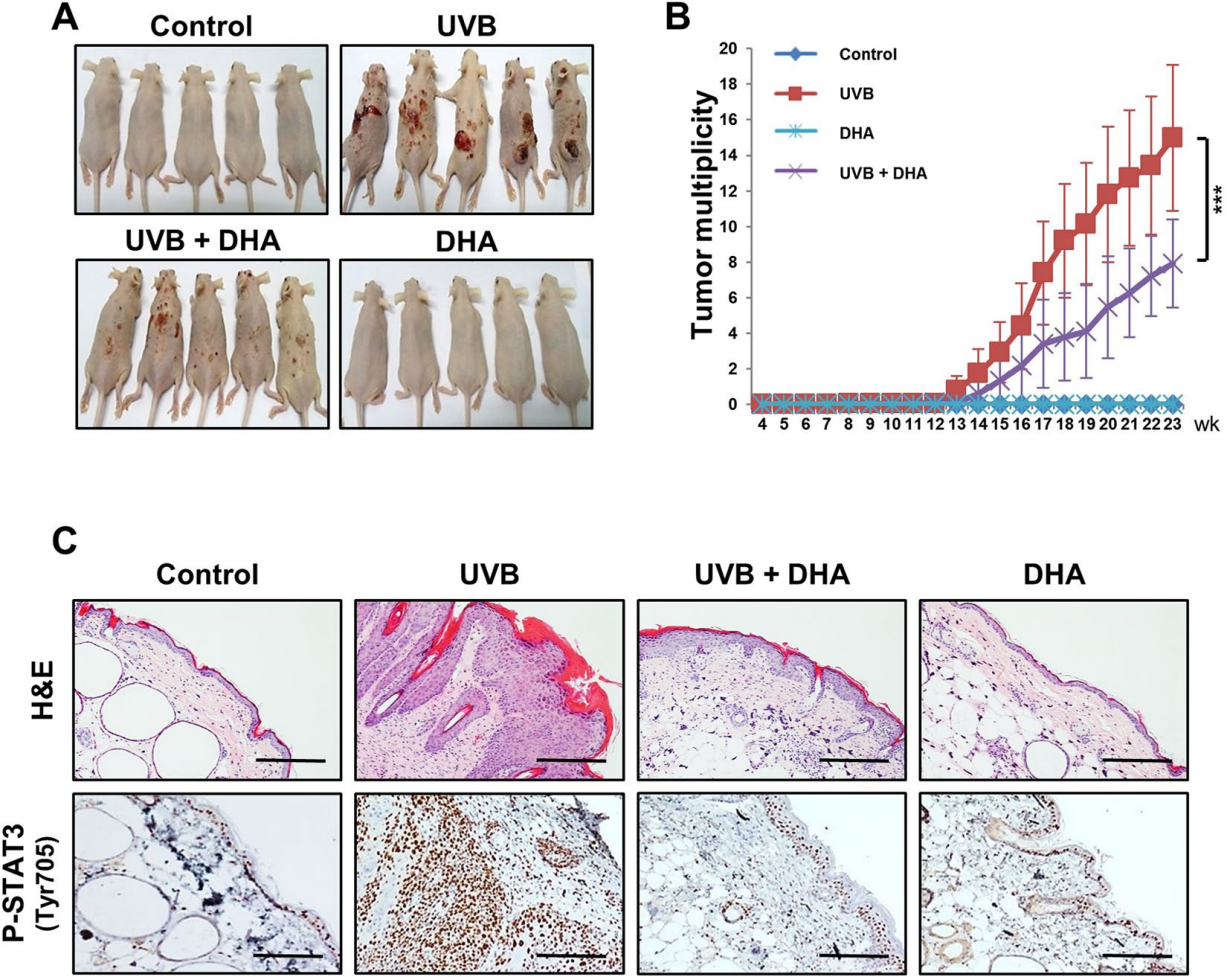
DHA inhibited UVB-induced papillomagenesis in hairless mouse skin. Female hairless mice (*n* = 15 per treatment group) were topically treated on their backs with DHA (10 µmol) 30 min prior to UVB radiation (180 mJ/cm^2^) three times a week until termination of the experiment at the 23^rd^ week. (**A**) Representative photographs of animals from different treatment groups. (**B**) The average number of papillomas per mouse in different treatment groups. Results are expressed as means ± SD. ***p < 0.001 versus UVB-irradiated control group. (**C**) Formalin-fixed skin tissues of mice were subjected to H&E staining and immunohistochemical analysis using a specific antibody to detect STAT3 phosphorylation at Tyr^705^. Magnifications, ×200. Scale bar is 100 µm.

*fat-1* mice have a substantially enhanced proportion of ω-3 PUFAs in which an increase in the DHA production is most prominent (Table 1). So, we examined the effect of repeated topical application of DHA on UVB-induced skin carcinogenesis in normal hairless mice. Figure 3A shows that UVB-induced skin papilloma formation was diminished by DHA pretreatment. Repeated topical application of DHA prior to UVB exposure significantly reduced the number of skin tumors per mouse (Fig. 3B). The inflammatory skin damage, as evidenced by H&E staining of skin papillomas, was ameliorated in the DHA-pretreated mice (Fig. 3C, *upper*). DHA treatment also significantly dampened the phosphorylation of STAT3 at the Tyr^705^ in the skin papillomas (Fig. 3C, *lower*).

### UVB-induced oxidative skin damage was attenuated in *fat-1* transgenic and DHA-treated mice

**Figure 4:**
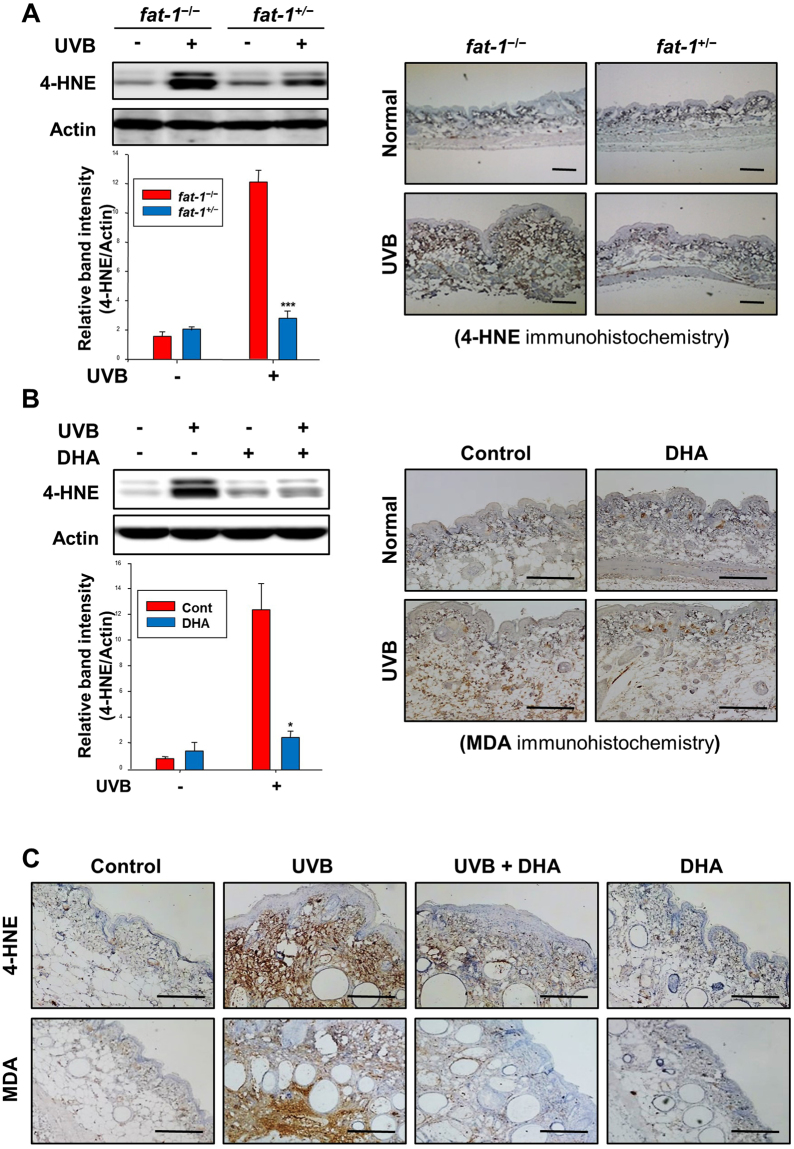
UVB-induced oxidative skin damage was attenuated in *fat-1* transgenic and DHA-treated WT mice. (**A,B**) Isolated protein extracts were electrophoresed and transferred to a PVDF membrane, which was probed using antibody for 4-HNE. Results are expressed as means ± SE (*n* = 5 per treatment group). ***p < 0.001 versus UVB-irradiated WT mice; *p < 0.05 versus UVB-irradiated control group. Formalin-fixed and paraffin-embedded tissues from UVB-irradiated mice were immunostained for detecting the 4-HNE (upper panels)- or MDA (bottom panels)-modified protein expression. Magnifications, ×100 (4-HNE) and ×200 (MDA). Scale bar is 100 µm. (**C**) Animal treatment and other experimental details are same as described in the legend to Fig. 3. The respective sections were immunostained for 4-HNE and MDA, and counterstained with hematoxylin. Positive 4-HNE and MDA staining yielded a brown-colored product. Magnifications, ×200. Scale bar is 100 µm.

Multiple lines of evidence suggest that UVB-induced generation of ROS is responsible for skin hyperplasia^28^ as well as lipid peroxidation^29^. 4-Hydroxy-2-nonenal (4-HNE) and malondialdehyde (MDA) are representative lipid peroxidation products^30,31^. There was lesser 4-HNE-induced protein modification in the hairless *fat-1* mouse skin as compared to WT one upon acute UVB irradiation (Fig. 4A). Topical application of DHA also attenuated UVB-induced accumulation of 4-HNE- and MDA-modified proteins in mouse epidermis (Fig. 4B). Moreover, repeated exposure to UVB up to 23 weeks increased the 4-HNE-modified protein expression, which was blunted in the skin of DHA-pretreated (Fig. 4C, *upper*) mice. Likewise, repeated topical application of DHA prior to UVB irradiation reduced the UVB-induced accumulation of MDA-modified protein in mouse skin papillomas (Fig. 4C, *lower*).

### The Nrf2-mediated induction of cytoprotective gene expression was elevated in the skin of *fat-1* and DHA-treated mice

**Figure 5:**
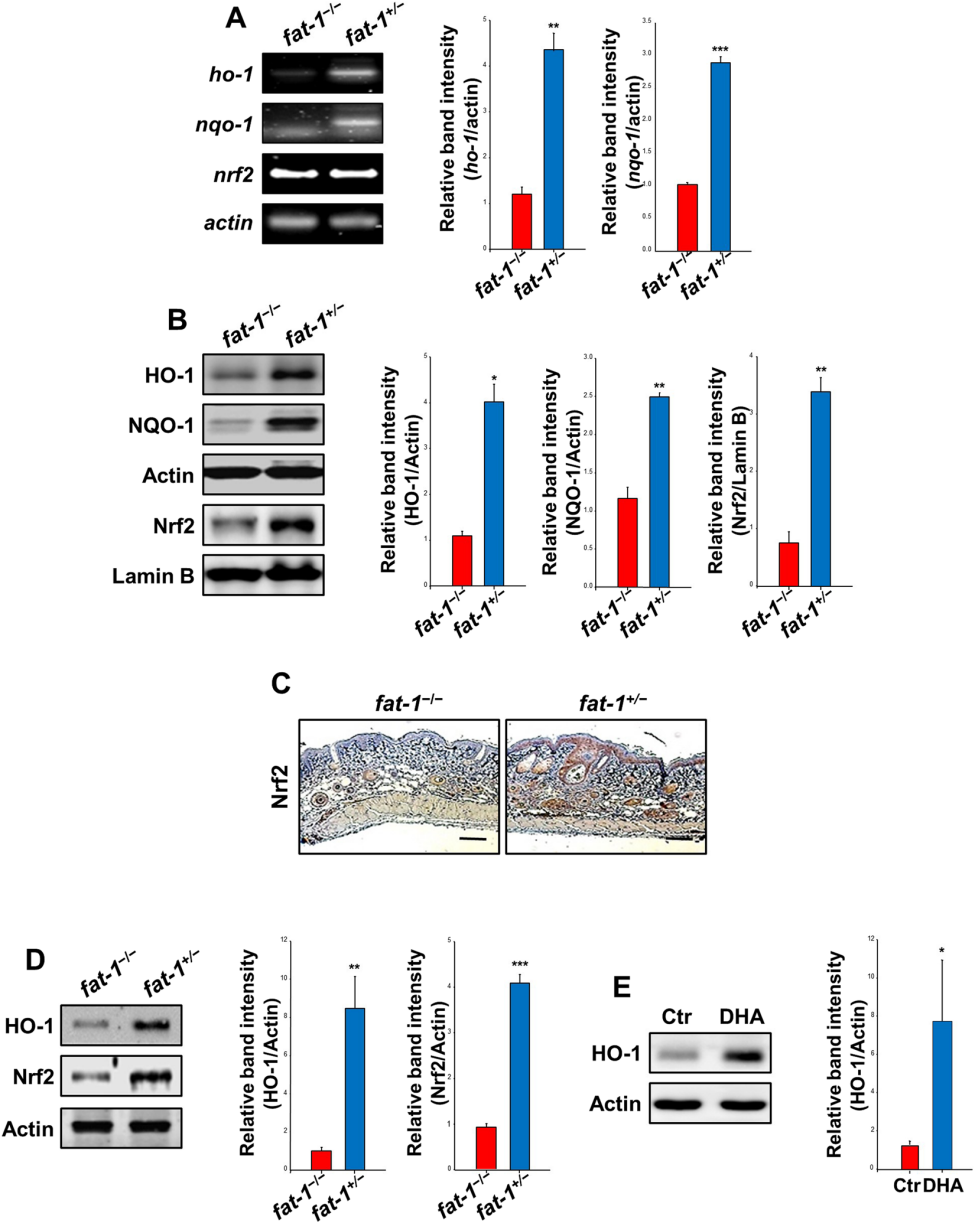
The Nrf2-mediated induction of cytoprotective gene expression was elevated in the skin tissues of *fat-1* transgenic and DHA-treated mice. Hairless *fat-1* transgenic and WT mice (*n* = 5 per treatment group) were maintained on AIN-93 diet supplemented with 10% safflower oil (rich in ω-6 fatty acids) for 5 weeks (**A**–**C**). (**A**) RT-PCR analysis was conducted to measure the mRNA levels of *ho-1, nqo1* and *nrf2*. (**B**) Collected skin tissues were placed on ice, and fat was removed to get an epidermal layer. The epidermal lysates and nuclear extracts from different groups were subjected to electrophoresis on SDS-PAGE and immunoblotted to detect protein expression of HO-1, NQO1 and Nrf2. (**C**) Paraffin-embedded skin tissue blocks were analyzed by IHC and the levels of Nrf2 (brown spots) were compared between hairless *fat-1* transgenic and WT mice. Magnifications, ×100. Scale bar is 100 µm. (**D**) After 23 weeks of feeding the 10% safflower oil diet, whole tissue extracts (30 μg protein) were analyzed for the protein levels of Nrf2-regulated antioxidant gene by immunoblotting. Data are expressed as means ± SE. *p < 0.05, **p < 0.01 and ***p < 0.001 versus WT mice. (**E**) Dorsal skin of female HR-1 hairless mice (*n* = 5 per treatment group) were topically treated with DHA (10 µmol). Control animals were treated with acetone in lieu of DHA. After treatment for 23 weeks, epidermal tissue lysates (30 μg protein) were subjected to electrophoresis on 10% SDS-polyacrylamide gel and immunoblotted to detect the level of HO-1 protein expression. *p < 0.05 versus the control group.

To investigate whether the decreased number of papillomas formed by UVB irradiation is attributable to augmentation of cellular defense capacity that counteracts excessive ROS accumulation, we analyzed the antioxidant gene expression in the skin of *fat-1* and DHA-treated mice. We found a robust enhancement in the expression of two representative cytoprotective enzymes HO-1 and NQO1, at both transcriptional and translational levels, in the *fat-1* mice fed diet containing 10% safflower oil rich in ω-6 PUFAs (Fig. 5A and B). Nrf2 plays a fundamental role in transcriptional activation of genes encoding HO-1 and NQO1. Elevated accumulation of Nrf2 was observed in the skin of *fat-1* mice (Fig. 5B and C). However, no significant change in the epidermal level of Nrf2 mRNA was found in the *fat-1* mice compared with their respective age-matched skin of WT mice (Fig. 5A). Consistently, the stabilization of Nrf2 protein and upregulation of its target protein, HO-1 were observed in the hairless *fat-1* mouse skin at the 23^rd^ week, as measured by Western blot analysis (Fig. 5D). In addition, repeated application of DHA thrice a week for 23 weeks induced the HO-1 expression in mouse skin (Fig. 5E).

### UVB-induced inflammation and oxidative stress were less severe in mouse embryonic fibroblasts (MEFs) from *fat-1* mice than those from WT animals

**Figure 6:**
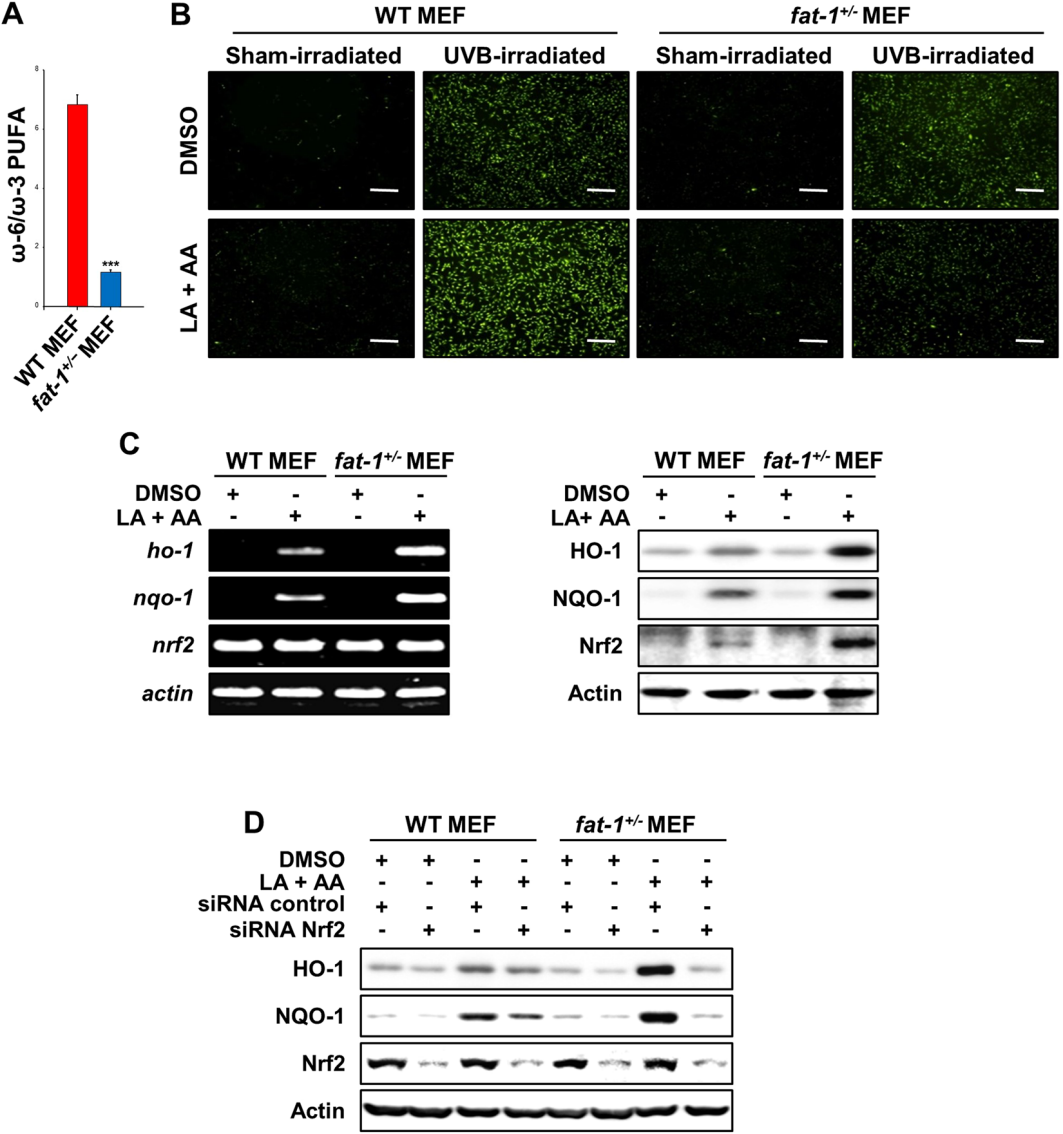
UVB-induced inflammation and oxidative stress were less severe in MEFs from *fat-1* transgenic mice than those in WT MEFs. At 4 weeks of age, male *fat-1* transgenic and female WT littermates were fed the 10% safflower oil and maintained throughout mating, pregnancy, and lactation on the same diet. Embryos were isolated from those mice and used to prepare fibroblasts. (**A**) PUFA profiles of MEFs were attained by using gas chromatography. A significantly decreased ratio of ω-6 to ω-3 fatty acids was observed in MEFs derived from *fat-1* mice compared with those in WT MEFs. Results are expressed as means ± SE. ***p < 0.001 versus WT MEFs. (**B**) MEFs were incubated with LA and AA (50 μM each) for 24 h before exposure to UVB radiation (20 mJ/cm^2^). After 1 h of UVB irradiation, cells were subjected to the DCF-DA assay. Enhancement of UVB-induced intracellular ROS generation was observed in the absence of *fat-1* gene. Magnifications, ×100. Scale bar is 100 µm. (**C**) MEFs were incubated with or without LA and AA (50 μM each) for 24 h, and their expression of *nrf2* and its target genes was assessed by RT-PCR. (**D**) MEF cells were transiently transfected with *nrf2* siRNA or control siRNA for 48 h and incubated with or without LA and AA (50 μM each) for 24 h. The expression of HO-1 and NQO-1 as well as Nrf2 was measured by Western blot analysis.

To further confirm the *in vivo* findings, MEFs were isolated from both WT and *fat-1* transgenic mice. A significantly lower ω-6/ω-3 fatty acid ratio was observed in *fat-1* MEFs as compared to WT MEFs (Fig. 6A and Table 2). Reduced levels of ω-6 PUFAs, especially linoleic acid (LA) and arachidonic acid (AA), were found in the *fat-1* MEFs as compared to the WT MEFs. However, the reduction of ω-6 PUFA levels in the *fat-1* MEFs was not as dramatic as increases in ω-3 fatty acids. To elucidate the protective role of the *fat-1* transgene in UVB-induced oxidative insults, MEFs were incubated with two representative ω-6 PUFAs, LA and AA, followed by UVB irradiation. As illustrated in Fig. 6B, UVB-irradiated MEFs showed an enormous increase in the ROS accumulation as compared to control MEFs. The ROS generation was further enhanced in the *fat-1* deficient MEFs incubated with ω-6 PUFAs, LA and AA. However, the decreased ω-6/ω-3 fatty acid ratio blocked UVB-induced ROS overproduction in the *fat-1* MEFs. The *fat-1* MEFs exhibited upregulation of the two representative antioxidant enzymes, HO-1 and NQO1, at both transcriptional and translational levels, to a greater extent than WT MEFs upon treatment with LA plus AA (Fig. 6C). Further, siRNA knock down of *nrf2* gene abrogated the expression of HO-1 and NQO1 in *fat-1* MEFs (Fig. 6D). This finding indicates that Nrf2 is essential for the induction of cytoprotective gene expression in *fat-1* MEFs.

**Table 2:** PUFA composition in embryonic fibroblasts from *fat-1* transgenic and WT mice.

		WT MEF	*fat-1* ^+/−^ MEF
Linoleic acid	C18:2	2.95 ± 0.08	1.51 ± 0.01^c^
γ-Linolenic acid	C18:3	0.07 ± 0.01	0.03 ± 0.01^a^
Arachidonic acid	C20:4	6.24 ± 0.13	3.89 ± 0.06^c^
ω-6 PUFAs (%)		9.26 ± 0.21	5.44 ± 0.05^c^
α-Linolenic acid	C18:3	0.17 ± 0.01	0.37 ± 0.03^b^
Eicosapentaenoic acid	C20:5	0.44 ± 0.01	1.56 ± 0.21^b^
Docosahexaenoic acid	C22:6	0.74 ± 0.02	2.67 ± 0.22^c^
ω-3 PUFAs (%)		1.35 ± 0.02	4.60 ± 0.08^c^
ω-6/ω-3 PUFAs		6.85 ± 0.10	1.19 ± 0.01^c^
ω-3/ω-6 PUFAs		0.15 ± 0.003	0.85 ± 0.01^c^

The levels of representative PUFAs were measured as described in Methods.

Data are expressed as means ± SE. (*n* = 3); ^a^ *p* < 0.05, ^b^ *p* < 0.01 and ^c^ *p* < 0.001 versus WT MEFs.

### DHA increased the stability of Nrf2 protein in mouse epidermal JB6 cells

**Figure 7:**
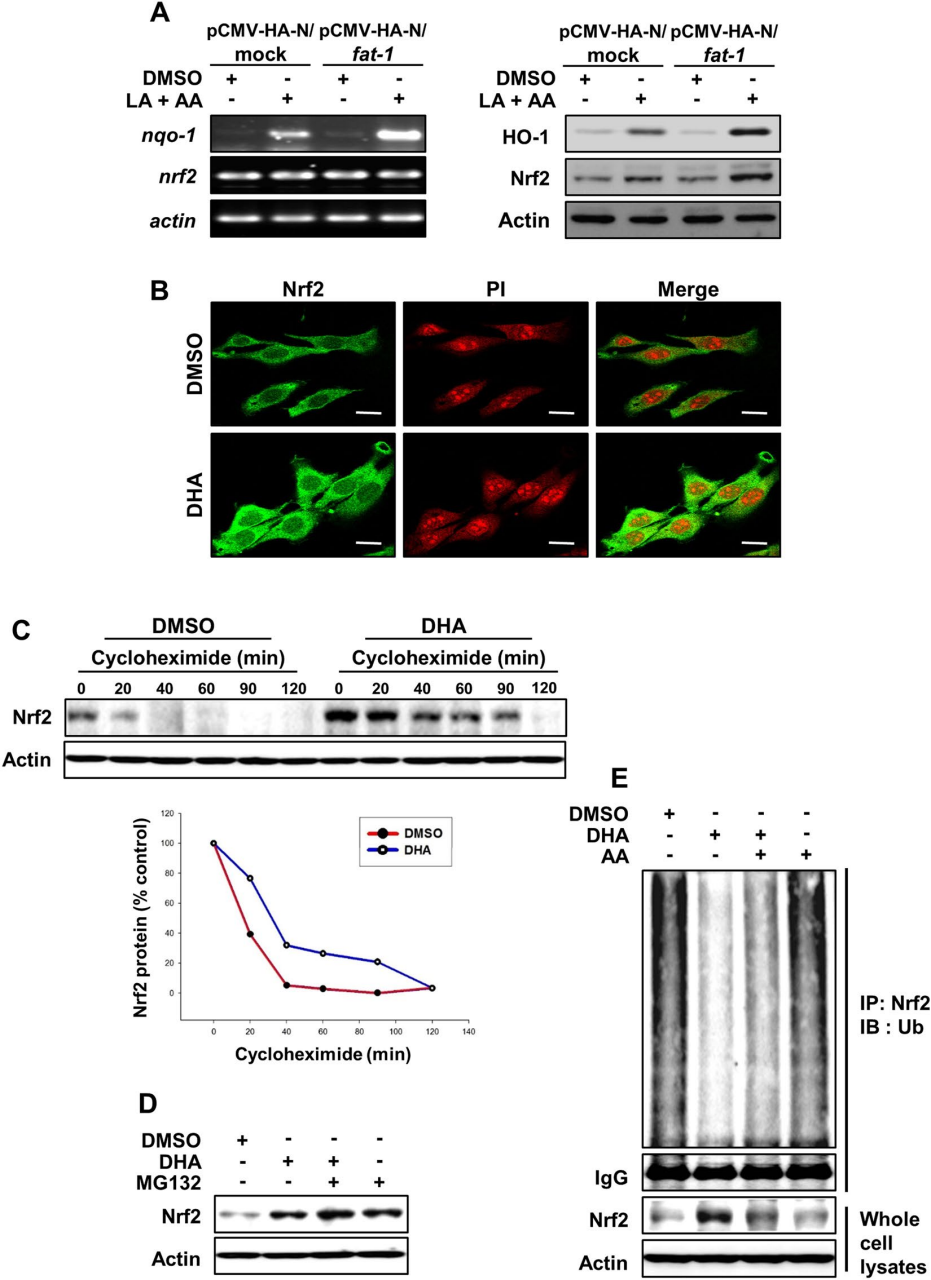
DHA increased the stability of Nrf2 protein in mouse epidermal JB6 cells. (**A**) JB6 cells were transfected transiently for 24 h with expression plasmids for pCMV-HA-mock or pCMV-HA-*fat-1*. Transfected cells were treated with or without LA and AA (25 μM) for 48 h prior to the RT-PCR and Western blot analysis to determine the levels of Nrf2 and cytoprotective proteins as as well as their mRNA transcripts. Transfection with pCMV-HA-*fat-1* increased protein expression of Nrf2 without altering its mRNA transcriptional level in JB6 cells. (**B**) JB6 cells were treated with DHA (50 μM) for 6 h, and nuclear localization of Nrf2 was determined by immunocytochemistry according to the protocol described under Materials and Methods. Magnifications, ×400. Scale bar is 20 µm. JB6 cells were challenged with 0.1 mg/mL cycloheximide (**C**) for the indicated times or 20 μM of MG-132 (**D**) for 30 min in the absence or presence of 50 μM of DHA for 6 h. Control cells were treated with DMSO alone. (**E**) After treatment of JB6 cells with DHA (50 μM) in the absence or presence of AA (25 μM) for 6 h, 500 μg of proteins was immunoprecipitated with anti-Nrf2 antibody and visualized by Western blot analysis with anti-ubiquitin antibody.

In another experiment, mouse epidermal JB6 cells were transfected with either pCMV-HA-control vector or pCMV-HA-*fat-1* vector. Overexpression of *fat-1* gene increased the Nrf2-mediated expression of cytoprotective enzymes (Fig. 7A). In contrast to the elevated Nrf2 protein expression, JB6 cells transiently transfected with pCMV-HA-*fat-1* vector exhibited no significant alteration in the steady state level of its mRNA transcript (Fig. 7A). We also examined the effect of DHA on the nuclear localization of Nrf2 in cultured mouse epidermal JB6 cells. As shown in Fig. 7B, DHA increased the accumulation of Nrf2 in the nucleus, which was confirmed by immunocytochemical analysis. To determine whether DHA stabilizes Nrf2 protein without inducing *de novo* synthesis, we monitored the degradation of basal and DHA-induced Nrf2 protein in JB6 cells after inhibition of protein synthesis by cycloheximide. As illustrated in Fig. 7C, Nrf2 protein in untreated control cells rapidly degraded after addition of cycloheximide, whereas Nrf2 in the DHA-treated JB6 cells underwent degradation more slowly. Many labile regulatory proteins, including signal-activated transcription factors, are commonly degraded by the 26S proteasomes. Treatment of JB6 cells with the proteasome inhibitor, MG-132 significantly increased the level of Nrf2 (Fig. 7D). Moreover, most proteins subjected to proteasomal degradation are marked by prior, covalent ligation of ubiquitin molecules to the ε-amino group of specific lysine residues. A ubiquitination assay revealed that the appearance of ubiquitinated Nrf2 was decreased with a concomitant increase in Nrf2 accumulation in cells treated with DHA (Fig. 7E).

## Discussion

Mounting evidence from epidemiological and laboratory-based studies suggest that long chain ω-3 PUFAs possess a broad range of health beneficial properties^16^ and exert protective effects against oxidative stress-induced inflammation^32^, aging^33^, cancer^34^, etc. DHA, one of the major ω-3 PUFAs, has been reported to inhibit UVB-induced inflammatory and oxidative injuries of mouse skin^17^. Rahman *et al*. demonstrated that topical application of DHA inhibited UVB-induced expression of COX-2 and NAD(P)H oxidase-4, an enzyme involved in the generation of ROS, in hairless mouse skin^17^. In line with the above observation, we have found that topical application of DHA attenuates epidermal hyperplasia and reduces the accumulation of lipid peroxidation products, such as 4-HNE and MDA, in UVB-irradiated mouse skin. In addition, our study revealed for the first time that inhibition of UVB-induced mouse skin papillomagenesis by topically applied DHA. The inhibitory effects of DHA on the UVB-induced increase in 4-HNE- and MDA-modified protein levels in skin indicate that this ω-3 fatty acid protects against photocarcinogenesis by suppressing the ROS accumulation in epidermis in response to UVB irradiation. These findings are in good agreement with the previously reported anti-oxidative effects of DHA^35,36^.

The present study was intended to assess the chemopreventive effects of endogenously produced ω-3 PUFAs against photocarcinogenesis using *fat-1* transgenic mice without the need for exogenous supply. Homozygous mutations in hairless gene cause a permanent hair loss, referred to as alopecia in both humans and mice^37^. The hairless mice have been used historically in studying experimentally induced skin toxicity and carcinogenesis^38^. To avoid the tedious procedure of repeated removal of hair for long-term UVB irradiation, we have generated hairless *fat-1*transgenic mice by cross-breeding of male *fat-1* ^+/−^ transgenic mice with female SKH-1 hairless mice. We noticed that an inflammatory response to UVB irradiation was significantly alleviated in the hairless *fat-1* ^+/−^ mice compared with that in the WT mice, which is associated with their relative resistance to UVB-induced papilloma formation.

COX-2 has been identified as a molecular link between inflammation and tumor promotion^39^. Abnormally elevated expression of COX-2 was observed in mouse skin papillomas formed after chronic exposure to UVB^40,41^. In the present study, the irradiation of hairless mice with UVB (180 mJ/cm^2^) for 23 weeks resulted in a marked increase in COX-2 expression. Hairless *fat-1* ^+/−^ mice capable of spontaneously producing ω-3 PUFAs exhibited significantly reduced expression of COX-2 in their dorsal skin compared with WT mice, and were protected from photocarcinogenesis. These findings indicate that not only topically applied, but also systemically administered ω-3 PUFAs could be efficient for chemoprevention of photocarcinogenesis.

A critical role for STAT3 in promoting proliferation, metastasis and immune evasion of tumor cells has been shown in various pathophysiologic conditions^42^. Kim *et al*. reported that constitutive activation of STAT3 enhanced UVB-induced skin carcinogenesis^25^. Likewise, STAT3 activation promoted keratinocyte survival and proliferation in response to UVB radiation^43^. Thus, inhibition of aberrantly overactivated STAT3 signaling is considered as a pragmatic approach for achieving the prevention of skin cancer. In our study, the repeated UVB irradiation caused Tyr^705^ phosphorylation of STAT3 in the skin papillomas, and this was attenuated in *fat-1* transgenic mice and DHA-treated WT mice.

ROS-mediated oxidative stress is capable of fostering the development of various pathological conditions mainly by overwhelming skin homeostatic antioxidant defenses. Body protects against oxidative stress and maintains the optimal intracellular redox environment through activation of a battery of antioxidant enzymes^15^. In line with this notion, mice expressing wild type HO-1 exhibited the lower multiplicity of skin papillomas than did HO-1 knockout animals^44^. Likewise, NQO1-null mice were susceptible to experimentally induced skin tumorigenesis to a greater extent than wild type mice^45,46^. Nrf2 plays a fundamental role in regulating expression of many anti-oxidative and cytoprotective proteins including HO-1 and NQO1. Nrf2 knockout mice were more prone to develop chemically induced skin tumors^47^. In our present study, one of the notable changes accompanying a lower degree of oxidative stress as well as inflammation and the reduction of papilloma formation in the skin tissues of *fat-1* mice challenged with UVB irradiation was the Nrf2-mediated induction of cytoprotective gene expression.

Since *fat-1* mice exhibited pronounced constitutive induction of HO-1 and NQO1 as well as accumulation of Nrf2 in their skin, we explored the mechanistic basis for the anti-oxidative effects elicited by endogenously produced ω-3 PUFAs by utilizing the *fat-1* MEFs. The inhibition of HO-1 and NQO1 expression in the Nrf2 siRNA-transfected *fat-1* MEFs suggests that the expression of these anti-oxidative enzymes was induced mainly through upregulation of Nrf2 signaling in the *fat-1* mice. Furthermore, DHA enhanced nuclear translocation of Nrf2 through stabilization of this transcription factor by inhibiting ubiquitination and 26S proteasomal degradation in the JB6 cells.

In conclusion, the present study demonstrates that constitutive ω-3 fatty acid production in the *fat-1* mice and topical application of DHA in wild type mice protect against UVB-induced inflammatory and oxidative skin damage and papillomagenesis through suppression of STAT3 activation and upregulation of Nrf2-mediated cytoprotective gene expression. These results highlight the importance of maintaining adequate levels of intracellular ω-3 PUFA in the management of skin disorders which are often caused by persistent inflammation and oxidative stress.

## Methods

### Ethics statement

All animal experiments were conducted in accordance with the guidelines of the Institutional Animal Care and Use Committee (IACUC) at Seoul National University. The experimental protocol was approved by the Animal Experimental Ethics Committee, Seoul National University (authorization number SNU-130201-1 and SNU-140624-2).

### Development of an *in vivo* hairless *fat-1* model

Male transgenic *fat-1* ^+/−^ mice^23^ were backcrossed (at least five times) to female mice with Skh:hr-1 hairless background (Charles River Laboratories, Wilmington, MA). Animals were kept in standard cages under specific pathogen-free conditions and fed a special diet (10% safflower oil), high in ω-6 and scarce in ω-3 PUFAs. The offsprings were examined for the hairless phenotypes and genotyped for *fat-1* ^+/−^ heterozygosity using the Tissue-Direct^TM^ PCR Kit (Lamda Biotech, Inc. St. Louis, MO). The genotyping primers of *fat-1* were as follows: *fat-1*, 5′-CTG CAC CAC GCC TTC ACC AAC C-3′ (forward) and 5′-ACA CAG CAG CAG ATT CCA GAG ATT-3′ (reverse).

### Preparation and maintenance of MEFs

Male *fat-1* ^+/−^ and female WT mice were paired and the pregnancies were monitored. Embryos were obtained at the day 13.5 after paring and used to prepare fibroblasts after removing head, heart and legs. The tails of the embryos were used to confirm the *fat-1* ^+/−^ genotype by PCR, and the embryo bodies were minced into small pieces and cultured in high glucose Dulbecco’s modified Eagle’s medium (DMEM; Gibco BRL, Grand Island, NY) supplemented with 10% fetal bovine serum (FBS; Gibco BRL, Grand Island, NY) and kept at 37 °C with 5% CO_2_.

### Analysis of PUFA composition

The levels of representative ω-6 and ω-3 PUFAs were determined by using gas chromatography as described previously^48^. Peaks of resolved fatty acids were identified by comparison with the fatty acid methyl ester reference standards (Sigma Aldrich, St. Louis, MO), and percentage of areas for all resolved peaks was measured by using the ChemStation software (Agilent Technologies, Palo Alto, CA).

### JB6 Cl41 cell culture

The mouse epidermal cell line (JB6 Cl41) was obtained from the American Type Culture Collection (Manassas, VA). The cells were maintained in minimum essential medium (MEM) containing 5% FBS, 25 μg/mL gentamicin, 0.01% sodium pyruvate and 1.5 mg/mL sodium bicarbonate in an atmosphere of 95% air and 5% CO_2_ at 37 °C. MEM, FBS, and gentamicin were purchased from Gibco BRL (Grand Island, NY).

### Source and protocols of UVB irradiation

A Biolink BLX-312 UV crosslinker (Vilbert Lourmat, Marne-la-Valle´ e, France) was used to irradiate mouse skin (180 mJ/cm^2^ dose of UVB) and MEFs (20 mJ/cm^2^ dose of UVB). To evaluate the prophylactic effect of DHA (Cayman Chemical Co., Ann Arbor, MI), the irradiated hairless mice were applied topically with 200 μL of acetone or DHA (10 µmol) to the dorsal skin 30 min before exposure to UVB radiation. Dorsal skin samples were obtained for epidermal sheets 2 h later. In a skin papillomagenesis model, female hairless mice were irradiated with UVB (180 mJ/cm^2^) three times a week for 23 weeks. Mice were sacrificed, and their skin and tumors were collected for subsequent studies.

### Histological and immunohistochemical analyses of paraffin-embedded skin sections

Sections of harvested mouse skin were washed with phosphate-buffered saline (PBS) and fixed with 10% buffered formalin and embedded in paraffin. Each section (4 μm) was stained with H&E. The H&E stained sections were examined under light microscope to detect the presence of lesions. Immunohistochemistry (IHC) was performed to detect the protein expression. The sections were cut on silanized glass slides, deparaffinized three times with xylene and rehydrated through graded alcohol bath. For the detection of respective protein expression, slides were incubated with antibodies raised against P-STAT3 (Cell Signaling Technology, Beverly, MA), COX-2 (Cayman Chemical Co., Ann Arbor, MI), proteins modified with 4-HNE and MDA (JaICA, Nikken SEIL Co. Ltd., Shizuoka, Japan) and Nrf2 (Santa Cruz Biotechnology, Inc., Santa Cruz, CA), and then developed using horseradish peroxidase-conjugated secondary antibodies (rabbit or mouse) (Dako, Glostrup, Denmark). The peroxidase binding sites were detected by staining with 3,3′-diaminobenzidine tetrahydrochloride. Finally, counterstaining was performed using Mayer’s hematoxylin.

### Tissue lysis and protein extraction

The fat-free epidermis was immediately placed in liquid nitrogen and pulverized in mortar. The pulverized skin was homogenized on ice for 20 s with a polytron tissue homogenizer and lysed in 1 mL ice-cold lysis buffer [150 mM NaCl, 0.5% Triton-X 100, 50 mM Tris–HCl (pH 7.4), 20 mM ethylene glycol tetra-acetic acid, 1 mM dithiothreitol (DTT), 1 mM Na_3_VO_4_ and protease inhibitors, 1 mM phenylmethyl sulfonylfluoride (PMSF), and ethylenediaminetetraacetic acid (EDTA)-free cocktail tablet] followed by a periodical vortex for 30 min at 0 °C. In other studies, MEFs and JB6 C141 cells were incubated with LA, AA (Cayman Chemical Co., Ann Arbor, MI), cycloheximide (Sigma-Aldrich, St. Louis, MO) or MG132 (Enzo Life Sciences, Inc., Farmingdale, NY). The treated cells were harvested, washed with PBS and suspended in the lysis buffer as mentioned above for 1 h on ice. The lysates were centrifuged at 14,800 *g* for 15 min at 4 °C.

### Preparation of cytosolic and nuclear extracts

Scraped dorsal skin of mice was homogenized in 800 μL of hypotonic buffer A [10 mM 4-(2-hydroxyethyl)-1-piperazineethanesulfonic acid (HEPES, pH 7.8), 1.5 mM MgCl_2_, 10 mM KCl, 1 mM DTT, 0.1 mM EDTA and 0.1 mM PMSF]. To the homogenates was added 80 μL of 10% Nonidet P-40 (NP-40) solution, and the mixture was then centrifuged for 2 min at 14,800 *g*. The supernatant was collected as cytosolic fraction. The precipitated nuclei were washed once with 500 μL of buffer A plus 50 μl of 10% NP-40, centrifuged, resuspended in 200 μL of buffer C [50 mM HEPES (pH 7.8), 50 mM KCl, 300 mM NaCl, 0.1 mM EDTA, 1 mM DTT, 0.1 mM PMSF and 20% glycerol] and centrifuged for 5 min at 14,800 *g*. The supernatant containing nuclear proteins was collected and stored at −70 °C. In preparing cytosolic and nuclear extracts from JB6 cells, cells were gently washed with cold PBS, scraped, and centrifuged at 1,190 *g* for 5 min. Pellets were suspended in cold hypotonic buffer A [10 mM HEPES (pH 7.9), 1.5 mM MgCl_2_, 10 mM KCl, 0.2 mM DTT, 0.3 mM EDTA and 0.1 mM PMSF]. The lysates were incubated for 10 min on ice and then centrifuged at 14,800 *g* for 5 min at 4 °C. Supernatant was collected as cytosolic extract. The pellets were washed with hypotonic buffer and resuspended in hypertonic buffer C [20 mM HEPES (pH 7.8), 400 mM NaCl, 1 mM EDTA, 1 mM DTT, 0.5 mM PMSF and 25% glycerol] keeping on ice for 30 min during rocking followed by centrifugation at 14,800 *g* for 5 min. The supernatant containing nuclear proteins was collected.

### Western blot analysis

Cell lysates (30 μg protein) were boiled in sodium dodecyl sulfate (SDS) sample loading buffer for 5 min before electrophoresis on 8–15% SDS-polyacrylamide gel (SDS-PAGE). After transfer to a polyvinylidene difluoride (PVDF) membrane (Gelman Laboratory, Ann Arbor, MI), the blots were blocked with 5% fat-free dry milk in Tris-buffered saline containing 0.1% Tween 20 (TBST) for 1 h at room temperature. The membranes were incubated for 12–24 h at 4 °C with dilutions of primary antibodies against P-STAT3 (Cell Signaling Technology, Beverly, MA), lamin B, ubiquitin (Invitrogen, Carlsbad, CA), COX-2 (Cayman Chemical Co., Ann Arbor, MI), actin (Sigma Aldrich, St. Louis, MO), proteins modified with 4-HNE (JaICA, Nikken SEIL Co. Ltd., Shizuoka, Japan), HO-1 (Stressgen Biotechnologies Co., San Diego, CA), NQO1 (Abcam Inc., Cambridge, MA) and Nrf2 (Santa Cruz Biotechnology, Inc., Santa Cruz, CA). Blots were washed three times with TBST at 7 min intervals followed by incubation with 1:3000 dilution of respective horseradish peroxidase conjugated secondary antibodies (rabbit, mouse or goat) in 3% fat-free dry milk-TBST for 2 h at room temperature. The blots were rinsed again three times with TBST. The immunoblots were visualized with an enhanced chemiluminescent (ECL) detection kit (Amersham Pharmacia Biotech, Buckinghamshire, UK) and LAS-4000 image reader (Fujifilm, Tokyo, Japan).

### Reverse transcription-polymerase chain reaction analysis (RT-PCR)

Total RNA was isolated from mouse skin tissues using TRIzol^®^ reagent (Invitrogen, Carlsbad, CA). To generate the cDNA from RNA, 1 µg of total RNA was reverse transcribed with murine leukemia virus reverse transcriptase (Promega, Madison, WI) for 50 min at 42 °C and again for 15 min at 72 °C. About 1 μL of cDNA was amplified with a PCR mixture (HS Prime Taq 2X Premix, Daejeon, South Korea) in sequential reactions. The primers used for each RT-PCR reactions are as follows: *cox-2*, 5′-CTG GTG CCT GGT CTG ATG ATG-3′ and 5′-GGC AAT GCG GTT CTG ATA CTG-3′; *actin*, 5′-AGA GCA TAG CCC TCG TAG AT-3′ and 5′-CCC AGA GCA AGA GAG GTA TC-3′; *ho-1*, 5′-TAC ACA TCC AAG CCG AGA AT-3′ and 5′-GTT CCT CTG TCA GCA TCA CC-3′; *nqo1*, 5′-TCG GAG AAC TTT CAG TAC CC-3′ and 5′-TGC AGA GAG TAC ATG GAG CC-3′; *nrf2*, 5′-CCT CTG TCA CCA AGC TCA AGG-3′ and 5′-TTC TGG GCG GCG ACT TTA TT-3′ (forward and reverse, respectively). Amplification products were analyzed by 2–3% agarose gel electrophoresis, followed by staining with SYBR Green (Invitrogen, Carlsbad, CA) and photographed using fluorescence in LAS-4000 (Fujifilm, Tokyo, Japan).

### Measurement of intracellular ROS accumulation

The accumulation of ROS in UVB-irradiated MEFs was monitored using the fluorescence-generating probe 2′,7′-dichlorofluorescein diacetate (DCF-DA; Invitrogen, Carlsbad, CA). Treated cells were rinsed twice with Hanks’ balanced salt solution (HBSS, Cellgro, Herndon, VA) and loaded with 10 μM DCF-DA for 30 min in a 5% CO_2_ incubator to assess ROS-mediated oxidation of DCF-DA to the fluorescent compound DCF. Cells were washed twice with HBSS, suspended in the complete media and examined under a micromanipulator system (Leica Microsystems, Heidelberg, Germany).

### Transient transfection with Nrf2 siRNA

Nrf2 siRNA and Stealth^TM^ universal RNAi negative control duplexes were from Invitrogen (Carlsbad, CA). The target sequence for Nrf2 siRNA is as follow: forward 5′-AAG AGU AUG AGC UGG AAA AAC UU-3′and reverse 5′-GUU UUU CCA GCU CAU ACU CUU UU-3′. MEFs were seeded at a density of 4 × 10^4^ cells/mL in 60-mm petridishes and grown to 60–70% confluence. Nrf2 siRNA (20 μM) was transfected into MEFs with Lipofectamine^®^ RNAi-MAX reagent (Invitrogen, Carlsbad, CA) according to the manufacturer’s instructions. After 48-h transfection, cells were treated with LA (50 μM) and AA (50 μM) for additional 24 h, and cell lysates were prepared as described earlier.

### Construction of plasmid pCMV-HA-N-*fat-1*

The murine full-length *fat-1* was amplified by RT-PCR from the total RNA obtained from the tails of *fat-1* ^+/−^ mice with primers 5′-CAT GGA GGC CCG AAT TCG GAT GGT CGC TCA TTC CTC A-3′ (forward) and 5′- ATC CCC GCG GCC GCG GTA CCT TAC TTG GCC TTT GCC TT-3′ (reverse) and subcloned into HA-tagged-pCMV expression vector (Clontech Laboratories, Inc., Mountain View, CA) as *EcoRI*/*KpnI* fragment. One day before transfection, JB6 C141 cells were seeded at a density of 3 × 10^4^ cells/mL in a 60-mm dishes and grown to 40–50% confluence growth media without antibiotic at 37 °C in a humidified atmosphere of 5% CO_2_/95% air. *fat-1* overexpresion vector (50 nM) was transfected into JB6 Cl41 cells with Lipofectamine^®^ 2000 reagent (Invitrogen, Carlsbad, CA) according to the manufacturer’s instructions. After 24-h transfection, cells were treated with LA (25 μM) and AA (25 μM) for additional 48 h, and cell lysates were prepared as described earlier.

### Immunocytochemical analysis of Nrf2

JB6 Cl41 cells were plated on the 8-well chamber slide (0.5 × 10^4^ cells/well) and treated with DHA. Cells were fixed in 95% methanol for 10 min at −20 °C. After rinse with PBS containing 0.1% Tween 20 (PBST), cells were blocked overnight at 4 °C in fresh blocking buffer [PBST, pH 7.4, containing 5% bovine serum albumin (BSA)]. Dilution (1:100) of primary anti-Nrf2 antibody was made in PBST with 5% BSA, and cells were incubated overnight at 4 °C. After three washing steps with PBST, the cells were incubated with a fluoresceinisothiocyanate-conjugated goat anti-mouse IgG secondary antibody in PBST with 5% BSA for 1 h at room temperature. Cells were also stained with propodium iodide and rinsed with PBST. Stained cells were analyzed under a confocal microscope (Leica Microsystems, Heidelberg, Germany) and photographed.

### Immunoprecipitation

JB6 cells were treated with DHA and/or AA for 6 h and lysed in 250 mM sucrose, 50 mM Tris-HCl (pH 8.0), 25 mM KCl, 5 mM MgCl_2_, 1 mM EDTA, 2 μM NaF, 2 μM sodium orthovanadate, 1 mM PMSF and 10 mM N-ethylmaleimide. Total protein (500 μg) was subjected to immunoprecipitation by shaking with Nrf2 primary antibody at 4 °C for 24 h followed by the addition of 40 μL of 25% protein G-agarose bead slurry (Santa Cruz Biotechnology, Inc., Santa Cruz, CA) and additional shaking for 2 h at 4 °C. After centrifugation at 10,000 *g* for 1 min, immunoprecipitated beads were collected by discarding the supernatant and washed with cell lysis buffer. After final wash, immunoprecipitate was resuspended in 50 μL of 2X SDS electrophoresis sample buffer and boiled for 5 min. Forty five-μL of supernatant from each sample was loaded on SDS-PAGE. The ubiquitinated Nrf2 was visualized by antibody against ubiquitin (Life Technologies, Carlsbad, CA).

### Statistical analysis

Except for the data on the tumor multiplicity expressed as the mean ± standard deviation (SD), all other values were expressed as the mean ± standard error (SE) of at least three independent experiments. Statistical significance was determined by Student’s *t*-test and a *p-*value of less than 0.05 was considered to be statistically significant.

### Data availability

All data generated or analysed during this study are included in this published article and its Supplementary Information files.

## Electronic supplementary material

Supplementary Information
