# Supplementary material for: Endogenous ω-3 Fatty Acid Production by fat-1 Transgene and Topically Applied Docosahexaenoic Acid Protect against UVB-induced Mouse Skin Carcinogenesis
Source: Sci Rep. 2017 Sep 14;7:11658. doi: 10.1038/s41598-017-11443-2 (PMC5599646; doi:10.1038/s41598-017-11443-2)
Supplement: Supplementary file 1 — Supplementary Information [file 41598_2017_11443_MOESM1_ESM.pdf]

Supplementary Information

Endogenous ω-3 Fatty Acid Production by *fat-1* Transgene and Topically Applied Docosahexaenoic Acid Protect against UVB-induced Mouse Skin Carcinogenesis

Hye-Won Yum[1,2], Jin Park[1,3], Hyun-Jung Park[4], Jun Wan Shin[1,2], Yong-Yeon Cho[5], Su-Jung Kim[3], Jing X. Kang[6] and Young-Joon Surh[1,2,3,4]

[1]Tumor Microenvironment Global Core Research Center, College of Pharmacy, Seoul National University, Seoul, 08826, South Korea

[2]Research Institute of Pharmaceutical Sciences, College of Pharmacy, Seoul National University, Seoul, 08826, South Korea

[3]Department of Molecular Medicine and Biopharmaceutical Sciences, Graduate School of Convergence Sciences and Technology, Seoul National University, Seoul, 08826, South Korea

[4]Cancer Research Institute, Seoul National University, Seoul, 110-744, South Korea

[5]Integrated Research Institute of Pharmaceutical Sciences, College of Pharmacy, The Catholic University of Korea, Bucheon, Gyeonggi-do, 420-743, South Korea

[6]Laboratory for Lipid Medicine and Technology, Massachusetts General Hospital, Harvard Medical School, Boston, MA, 02129, USA

a

Fig. 1D

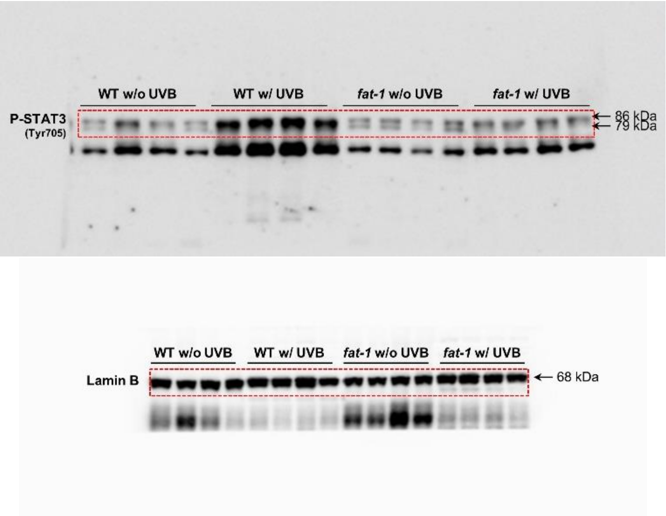

b

Fig. 2C

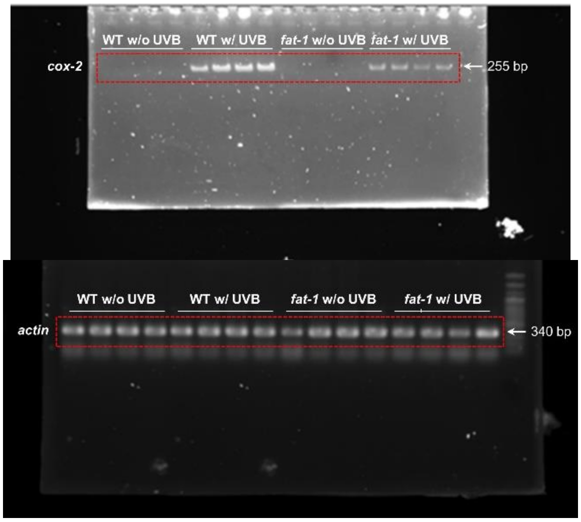

c

Fig. 2D

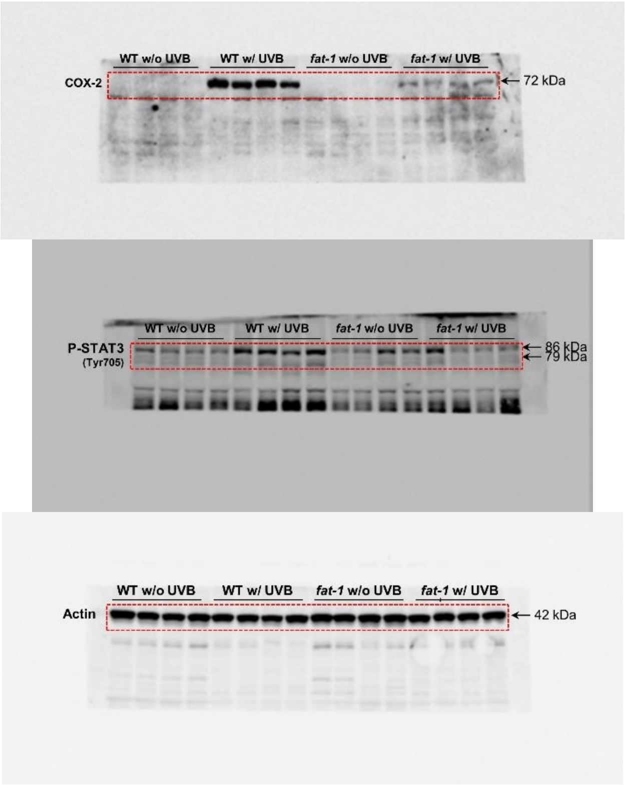

d

Fig. 4A

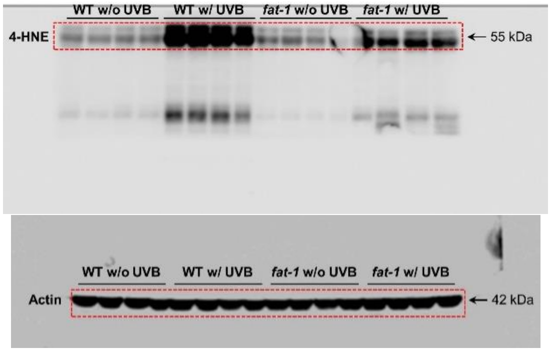

e

Fig. 4B

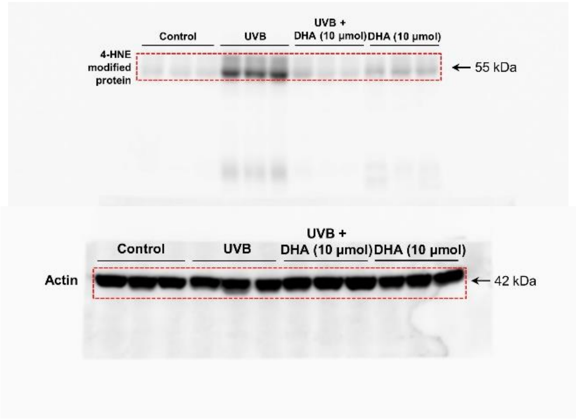

f

Fig. 5A

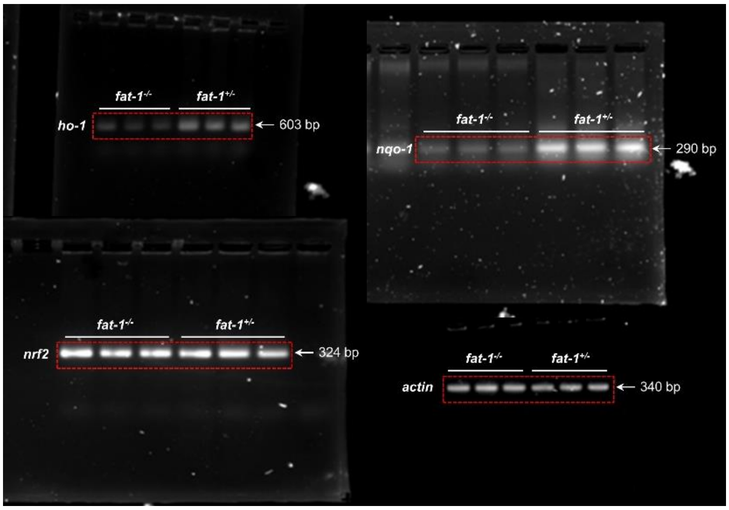

g

Fig. 5B

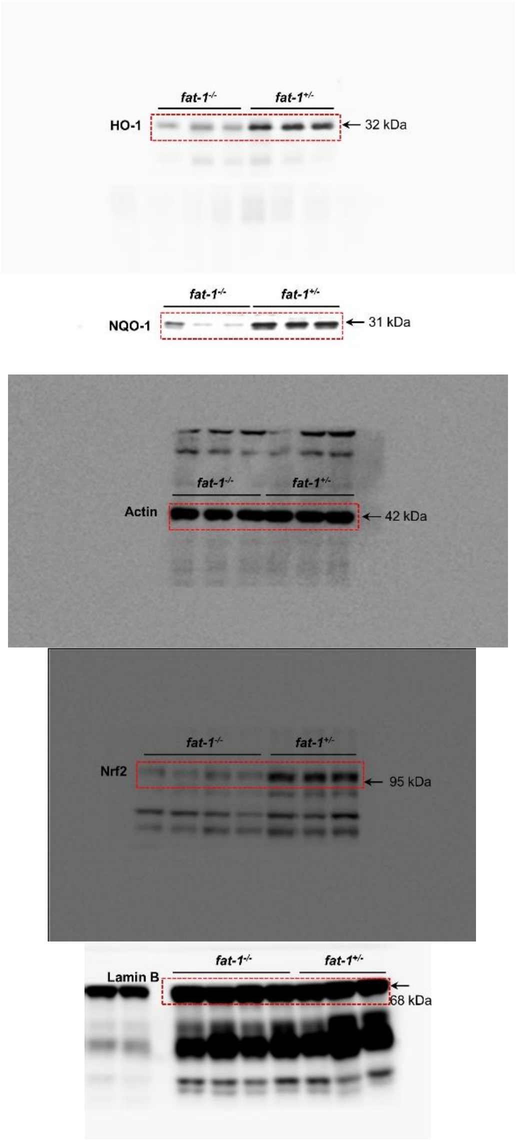

h

Fig. 5D

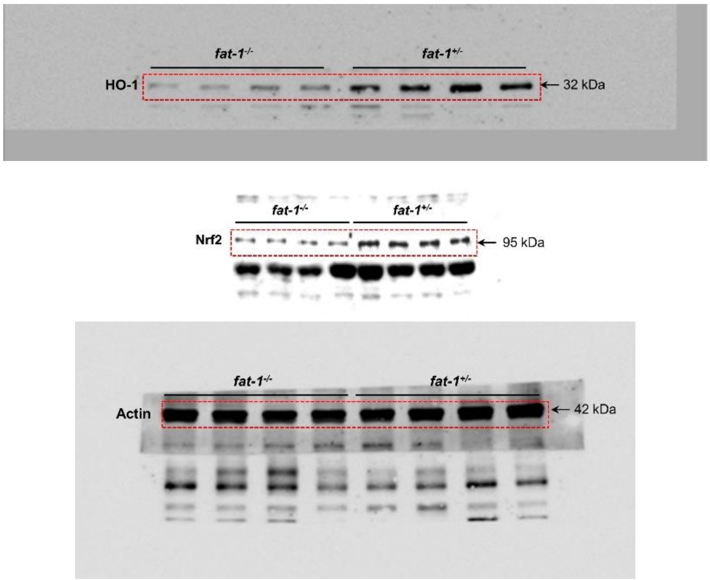

i

Fig. 5E

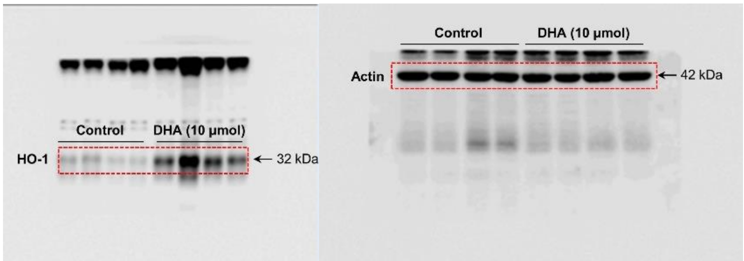

j

Fig. 6C

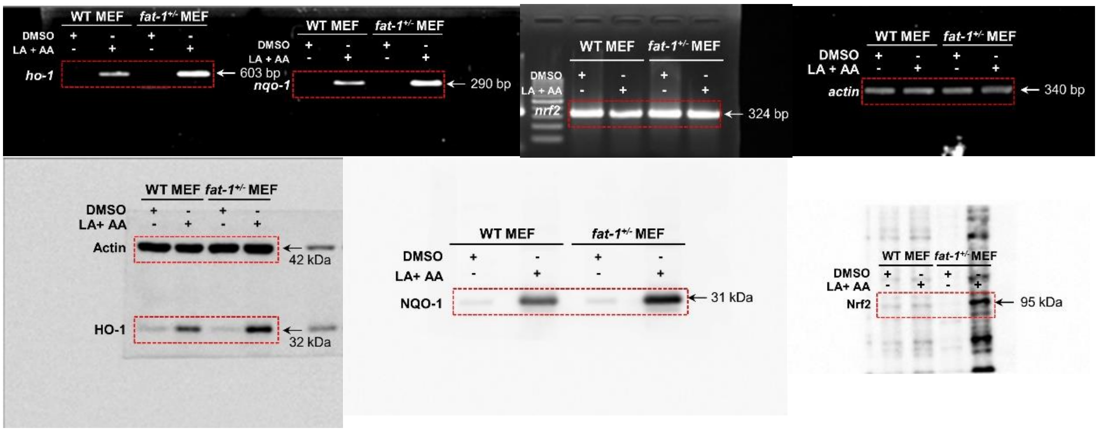

k

Fig. 6D

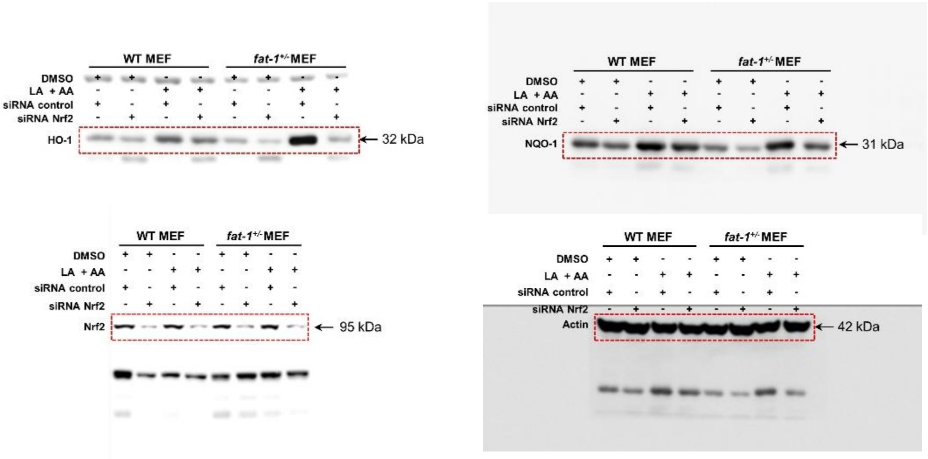

l

Fig. 7A

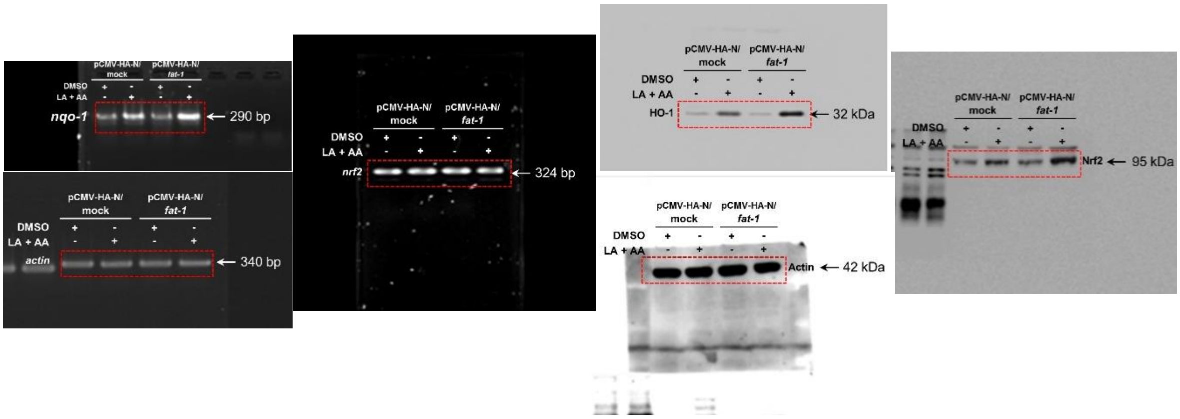

m

Fig. 7C

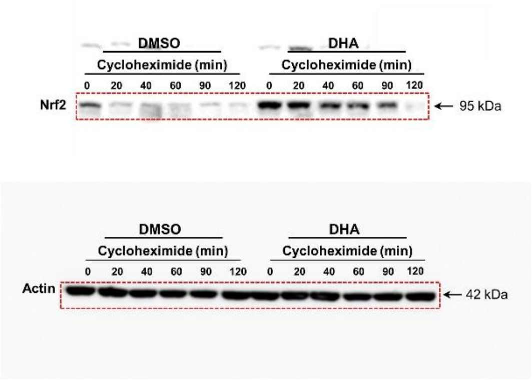

n

Fig. 7D

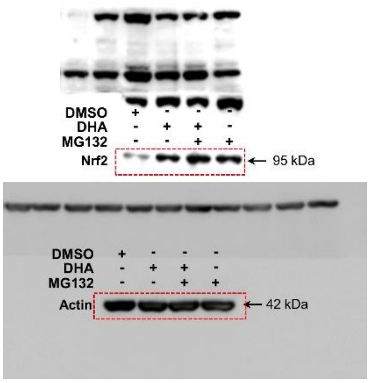

o

Fig. 7E

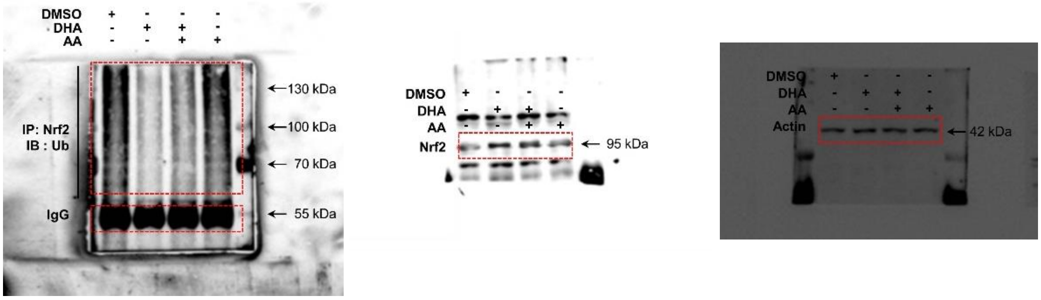

Supplementary Figure. Full-length gels and western blots. (**a**) Full-length blots corresponding to Fig. 1. Indicated part (red box) or bands (arrows) are shown in Fig. 1D. (b to c) Full length gels and blots corresponding to Fig. 2. Indicated part (red box) or bands (arrows) are shown in Fig. 2C (**b**) and Fig. 2D (**c**). (d to e) Full-length blots corresponding to Fig. 4. Indicated parts (red boxes) or bands (arrows) are shown in Fig. 4A (**d**) and Fig. 4B (**e**). (f and i) Full length gels and blots corresponding to Fig. 5. Indicated part (red box) or bands (arrows) are shown in Fig. 5A (**f**), Fig. 5B (**g**), Fig. 5D (**h**) and Fig. 5E (**i**). (j to k) Full-length gels and blots corresponding to Fig. 6. Indicated part (red box) or bands (arrows) are shown in Fig. 6C (**j**) and Fig. 6D (**k**). (l to o) Full-length blots and gels are corresponding to Fig. 7. Indicated part (red box) or bands (arrows) are shown in Fig. 7A (**l**), Fig. 7C (**m**), Fig. 7D (**n**) and Fig. 7E (**o**).
